# Supplementary material for: MetaRibo-Seq measures translation in microbiomes
Source: Nat Commun. 2020 Jun 29;11:3268. doi: 10.1038/s41467-020-17081-z (PMC7324362; doi:10.1038/s41467-020-17081-z)
Supplement: Supplementary file 10 — Supplementary Data 7 [file 41467_2020_17081_MOESM10_ESM.zip › File2/Confidence_High_Taxonomy/133166_out.krona.html]

Javascript must be enabled to view this page.

members
magnitude
magnitudeUnassigned
count
unassigned
taxon
rank

133166\_out

51

superkingdom
2
51

1239
2
phylum

class
2
186801

2
186802
order

family
1
541000

genus
1
1263

1
2293207
species

SRS103987\_contig\_number\_2700

family
1
31979

genus
1
1485

1262806
1
species

SRS064757\_contig\_number\_22614

phylum
976
49

class
49
200643

order
171549
49

171552
49
family

genus
838
49

59823
4
species

SRS044535\_contig\_number\_contig-100\_3892.112923SRS050752\_contig\_number\_21082SRS103987\_contig\_number\_1429SRS1041134\_contig\_number\_1833

species

SRS012969\_contig\_number\_2123SRS012989\_contig\_number\_7179SRS013098\_contig\_number\_contig-100\_1628.301371SRS013638\_contig\_number\_9120SRS013687\_contig\_number\_19666SRS014415\_contig\_number\_1778SRS015590\_contig\_number\_4870SRS015782\_contig\_number\_contig-100\_1038.229456SRS015794\_contig\_number\_1519SRS016541\_contig\_number\_contig-100\_1714.1714SRS017375\_contig\_number\_4SRS018888\_contig\_number\_4593SRS019285\_contig\_number\_37SRS019910\_contig\_number\_contig-100\_213.140487SRS023526\_contig\_number\_contig-100\_1335.217200SRS023914\_contig\_number\_12192SRS024132\_contig\_number\_4759SRS043841\_contig\_number\_9224SRS045713\_contig\_number\_contig-100\_1244.124474SRS046502\_contig\_number\_12834SRS047044\_contig\_number\_16424SRS047433\_contig\_number\_18823SRS049712\_contig\_number\_3856SRS053398\_contig\_number\_17108SRS058145\_contig\_number\_contig-100\_729.35332SRS065397\_contig\_number\_4312SRS074586\_contig\_number\_4679SRS077194\_contig\_number\_21234SRS077641\_contig\_number\_2090SRS077849\_contig\_number\_23165SRS077991\_contig\_number\_51SRS078419\_contig\_number\_12209SRS097958\_contig\_number\_16695SRS104036\_contig\_number\_7602SRS1041137\_contig\_number\_contig-100\_1335.176756SRS104327\_contig\_number\_5090SRS105082\_contig\_number\_648SRS144297\_contig\_number\_495SRS144506\_contig\_number\_19823SRS144603\_contig\_number\_14821SRS144714\_contig\_number\_2962SRS146764\_contig\_number\_17770SRS147614\_contig\_number\_3809SRS148253\_contig\_number\_contig-100\_548.96956SRS150029\_contig\_number\_contig-100\_1956.93603
45
165179
